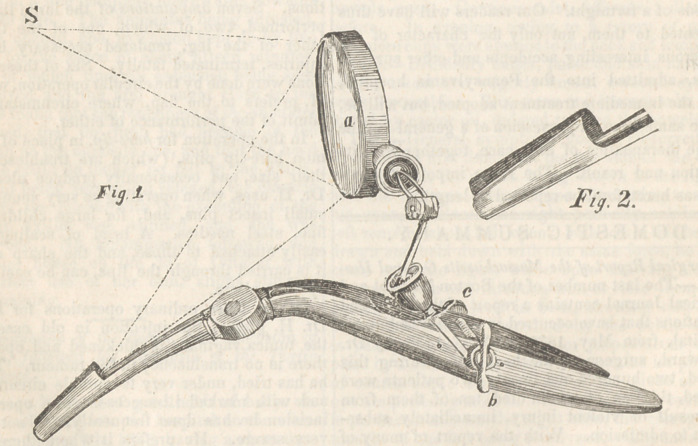# Domestic Summary

**Published:** 1838-10-10

**Authors:** 


### DOMESTIC SUMMARY.

*Surgical Report of the Massachusetts General Hospital.*—The last number of the Boston Medical and Surgical Journal contains a report of the cases and operations that have occurred in the Massachusetts hospital, from May, 1837, to May, 1838, by Dr. Hayward, surgeon to the hospital. During this period, two hundred and twenty-two patients were treated, thirteen of whom died, ten of them from the result of violent injury, immediately subsequent to admission. With the report of many of the cases, Dr. Hayward has incorporated some interesting remarks, of which we shall make a brief abstract.

*Erysipelas* has been and continues to be a great annoyance in the surgical wards of the Boston hospital; but, since an improvement in the ventilation of the establishment, during the past year, it has notably decreased, no death having occurred out of eight cases treated, and the character of the disease having been much less formidable, than in previous years. The treatment adopted in the hospital, consists of an active emetic, followed by a purgative, and this succeeded by some mild diaphoretic, during the first few days of the disease. At a very early period, quinine and other tonics, with a generous diet, are given with advantage. The only topical remedy employed is local bleeding; other applications have been found utterly inefficacious in arresting the disease, and those only are used which are most comfortable to the patient.

In *fracture of the lower jaw*, Dr. Hayward recommends a simple mode of treatment, which he has frequently found very efficacious. When there are teeth on each side of the fracture, and the bone is not comminuted, the ends of it can be kept in exact apposition, by passing a silver wire or strong thread around these teeth, and tying it tightly. This will be found a useful auxiliary in more severe cases, in which it may be required to use splints and bandages, or to insert a piece of cork between the jaws, as recommended by Delpech.

Four cases of *inflammation of the hernial sac* are reported. In the first, the sac which was gangrenous, was cut down upon and removed. The second was reduced by an active antiphlogistic course. The third suppurated, and, after the contents of the abscess were discharged, the sac contracted, but, though much reduced in size at the time of the patient's discharge, it could be plainly felt in its whole extent. Over the fourth case, the sudden reduction of the tumour throws some degree of obscurity.

Fifty-three operations were performed during the year, all of them successful, with two exceptions. Seven *amputations* of the large limbs were performed, two of which, one of the thigh, the other of the leg, rendered necessary by severe injuries, terminated fatally. Six of these amputations were done by the circular operation, which Dr. H. prefers to the flap, where circumstances will admit of the performance of either.

In the operation for *hare-lip*, in place of the common hare-lip pins, (which are troublesome from their size, and occasionally produce ulcerations,) Dr. H. uses, when operating on very young infants, small insect pins, and, for large children, long, fine, steel needles. A head of sealing wax is easily attached to these, and the sharp end, after it is carried through the lips, can be easily cut off by bone pliers.

Of the three ordinary operations for *hydrocele*, Dr. H. prefers the injection in old cases, where the tunica vaginalis is thickened and opaque, and there is no translucency in the tumour. The seton he has tried, under very favourable circumstances, and with marked ill-success. The operation by incision he has done frequently, and not found it very severe. He prefers it where there is any doubt as to the nature of the disease, and he operated in this manner in the case mentioned in his report. With acupuncturation he has been unsuccessful, but intends not to abandon it without further trial. In operating by injection, has the doctor ever employed a solution of iodine, which has been recommended by Velpeau, and since used with success in the Pennsylvania hospital?

In *prolapsus ani*, Dr. H. operates with the ligature, not feeling justified in exposing his patient to the risk of hæmorrhage from the knife.

*An improved Auriscope.* By JOHN T. SHARPLESS, M. D., of Philadelphia.

THE difficulty of minutely examining the recesses of the ear and nose is so great, that a suitable instrument to aid us in the investigation of the diseases of those cavities has been very desirable. Having, in vain, made every inquiry for an apparatus that would dilate these canals, and throw a stream of light to the bottom, I have been induced to contrive an arrangement that is extremely simple, and yet effective. The same construction may have been employed by others, and the Auriscope of Curtis may resemble it, for aught I know; but I know no one who has even seen that instrument, and, so far as I am acquainted, mine is entirely new in the most essential part. The valves of this speculum have long been used by others, but the handles have been generally placed at right angles with them, and the clothing of the neck of the patient has necessarily much interfered with their employment. The *direct* rays of the sun have also generally been admitted into the cavities. It was to remove the disadvantages of this mode of applying the light, that the present instrument was particularly designed. In the first place, when the sun is high in the heavens, the neck of the patient must be greatly strained to admit a *direct* ray into the ear, and the head must be turned almost downward to permit an illumination of the nose. These canals, moreover, are so narrow, that as the eye of the surgeon must be in the same line with the light, his head is a great obstruction to its course; and, indeed, when one is most successful in catching a sight of the tympanum, the eye must be so far from the object, that it becomes indistinct. The hand, also, during an operation, is another impediment both to the light and vision. In using a transparent lens to transmit and concentrate the rays, the same difficulty exists. The glare on the whole side of the face, etc., also renders the vision obscure.

The advantages of the present instrument are obvious. There is no dazzling of the eye by reflection from surrounding surfaces:—the head, no matter how vertical the sun, can be held nearly in a natural position, and the hand that holds the speculum rests upon the shoulder, away from the clothing of the neck. The *edges* of the entering end being slightly bent inward, enable the valves to pass far into the canal without scraping the delicate sides; and when dilated, do not press a sharp edge into the soft membrane. The closed blades being conical in their united form, when they are sufficiently separated to expose the tympanum, they become parallel to each other, and therefore have all the advantage thought to be gained by the right-angled handles. When the valves are not used, the mirror can be employed to the same advantage in throwing a beam of light to the bottom of the cavity, instead of the direct rays of the sun. The outsides of the valves should be highly polished, but the rest of the instrument, except the cavity of the blades, must be blackened. Perhaps this inner surface, being made dark, would render vision more distinct.

*Fig. 1.* *Fig. 2.*

*Fig.* 1. The instrument reduced about one-third in size. *a*, a concave mirror, one inch and a half in diameter, of about four inch focus, set into a brass frame, placed upon the extremity of an arm, with four joints. The first, *b*, is attached to the left handle, by a thumb screw. The second and fourth are ball and socket, with buckskin around the ball to prevent slipping; and the third is an ordinary hinge. *c*, a round thumb screw on the right handle, moving on a rod running through one handle and attached to the other, to retain the handles where they are placed by pressure of the fingers. The thread of this screw should be very fine, so that in adding a trifle to the dilatation, the change should be very gradual, which makes it much less painful. There should be a spring between the handles, to keep them closed, when entering. Every possible motion being possessed by the arm, the mirror can catch the sun, and cast a spectrum in any direction. When this is about one-fourth of an inch in diameter, which is the proper size, there is no disagreeable heat produced by the hottest summer's sun.

All the instruments for operation in these cavities can be made with long handles, so that the hand can be behind the mirror.

No. 2. The valves of their full size.—*Select Med. Lib. and Eclect. Journ. of Med.*, September, 1838.

---

*Select Medical Library.*—The October number completes the second volume of this publication. For the coming year, the following original works are announced:—On Yellow Fever, by a Physician of Philadelphia; on Orthopœdy, by Dr. Togno; a History of American Surgery, by Professor Dunbar; Ricord's Treatise on Venereal Diseases, translated by Dr. Biddle; on the Dengue Fever of the Southern States, by Professor Dickson; and an Essay by Dr. Dale on the question—Is Medical Science favourable to Scepticism? The lectures of Drs. Williams and Clutterbuck are to be republished.

---

*Southern Medical and Surgical Journal.*—We have received the October number, the first of the third volume of this journal. This is one of the best of our medical periodicals, and we learn with pleasure that it is no longer a matter of doubt that it can be sustained. The number before us, contains much valuable original matter.

---

The case of successful amputation of one half the lower jaw-bone, by Professor Paul F. Eve, which appeared in our nineteenth number, is, we notice, an original article in the South. Med. and Surg. Journal, for July. It was received in pamphlet form, before the Journal came to hand, and was not therefore credited to its original source.

---

*Capsicum in Dysentery.*—Dr. Thomas Miner, of Middletown, Conn., has addressed to the editor of the Boston Med. & Surg. Journal some remarks upon Dr. Gerhard's lecture on Dysentery, in which he says that: "Perhaps in almost every case of atonic dysentery, where opium, astringents, wine, alcohol, and other diffusible stimulants fail of producing their customary effects, *capsicum* is the best adjuvant that opium can have. Within the last few weeks, I have seen a formidable case of dysentery, apparently desperate, yield, within a day, to a pill of opium and capsicum, a grain each, given every two hours." Dr. Miner recommends the same combination in ataxic fevers, and, with the addition of one or two grains of the sugar of lead, in passive hemorrhage.

## Figures and Tables

**Fig. 1. Fig. 2. f1:**